# Precision Medicine in House Dust Mite-Driven Allergic Asthma

**DOI:** 10.3390/jcm9123827

**Published:** 2020-11-26

**Authors:** Ibon Eguiluz-Gracia, Francisca Palomares, Maria Salas, Almudena Testera-Montes, Adriana Ariza, Ignacio Davila, Joan Bartra, Cristobalina Mayorga, Maria Jose Torres, Carmen Rondon

**Affiliations:** 1 Allergy Unit, Hospital Regional Universitario de Malaga, 29009 Malaga, Spain; iboneguiluz@gmail.com (I.E.-G.); mariasalascassinello@hotmail.com (M.S.); a_testera_montes@hotmail.com (A.T.-M.); mayorga.lina@gmail.com (C.M.); mjtorresj@gmail.com (M.J.T.); 2 Allergy Research Group, Instituto de Investigación Biomedica de Malaga-IBIMA and ARADyAL, 29009 Malaga, Spain; francis.p.j@hotmail.com (F.P.); a.arizaveguillas@gmail.com (A.A.); 3 Department of Medicine and Dermatology, Universidad de Malaga, 29016 Malaga, Spain; 4 Allergy Department, University Hospital of Salamanca, 37007 Salamanca, Spain; idg@usal.es; 5 Allergy Research Group, Institute for Biomedical Research of Salamanca (IBSAL) and ARADyAL, 37007 Salamanca, Spain; 6 Department of Biomedical and Diagnostic Sciences, Universidad de Salamanca, 37007 Salamanca, Spain; 7 Allergy Section, Pulmonology, Hospital Clinic, Universitat de Barcelona, 08036 Barcelona, Spain; JBARTRA@clinic.cat; 8 Clinical & Experimental Respiratory Immunoallergy (IRCE), Instituto de Investigaciones Biomedicas Pi I Sunyer (IDIBAPS)-ARADyAL, 08036 Barcelona, Spain; 9 Laboratory for Nanostructures for the Diagnosis and Treatment of Allergic Diseases, Andalusian Center for Nanomedicine and Biotechnology (BIONAND), 29590 Malaga, Spain

**Keywords:** house dust mites, allergic asthma, local allergic asthma, bronchial allergen challenge, sublingual allergen immunotherapy

## Abstract

House dust mites (HDMs) are the allergenic sources most frequently involved in airway allergy. Nevertheless, not every sensitized patient develops respiratory symptoms upon exposure to HDM, and there is a clinical need to differentiate allergic asthmatics (AAs) from atopic non-allergic asthmatics with HDM sensitization. This differentiation sometimes requires in vivo provocations like the bronchial allergen challenge (BAC). Interestingly, recent data demonstrate that non-atopic patients with asthma can also develop positive BAC results. This novel phenotype has been termed local allergic asthma (LAA). The interest in identifying the allergic triggers of asthma resides in the possibility of administering allergen immunotherapy (AIT). AIT is a disease-modifying intervention, the clinical benefit of which persists after therapy discontinuation. Recently, new modalities of sublingual tablets of HDM immunotherapy registered as pharmaceutical products (HDM-SLIT tablets) have become commercially available. HDM-SLIT tablets have demonstrated a robust effect over critical asthma parameters (dose of inhaled corticosteroids, exacerbations, and safety), thus being recommended by international guidelines for patients with HDM-driven AA. In this review, we will summarize the current knowledge on the phenotype and endotype of HDM-driven AA, and LAA, address the difficulties for BAC implementation in the clinic, and discuss the effects of AIT in AA and LAA.

## 1. Introduction

Asthma is an inflammatory condition of the bronchial mucosa affecting 10% of children and 5% of adults in Western countries [1]. The disease imposes a high direct burden to health systems in medications, medical consultations, and hospitalizations [2]. Asthma is also associated with significant school and work absenteeism and presentism in children and adults [2]. Moreover, the condition is closely related to inflammatory diseases of the upper airways, further amplifying its impact [3].

Asthma is also a heterogeneous disease in phenotypes, evolution, and response to therapy [4]. Allergic asthma (AA) is the most frequent phenotype, and its prevalence is progressively increasing worldwide [5]. Among the different allergenic sources, house dust mites (HDMs) are the ones most commonly involved in airway allergy, including AA [6]. Nevertheless, asymptomatic HDM sensitization is also very frequent among healthy subjects and asthmatic patients [7]. Interestingly, recent data suggest that HDM can also trigger bronchial asthma in non-atopic individuals [8]. This new phenotype has been termed local allergic asthma (LAA). Of note, both AA and LAA are associated to nasal inflammatory diseases, which can be considered their counterparts in the upper airways. Therefore, as emphasized by the united airways concept, it would probably be more appropriate to use the terms atopic and local respiratory allergy. Regarding evolution, around 10% of asthma patients develop severe forms of the disease [5]. Despite not representing a majority of cases, severe asthma accounts for 80% of the costs attributable to the condition, mainly due to repeated exacerbations [2]. Allergens, especially those in the feces and bodies of HDM, are known triggers of asthma exacerbations [9], suggesting that allergic mechanisms are essential in severe asthma. Nevertheless, the role of allergy in severe asthma has been historically questioned [10], probably due to the difficulty of conducting bronchial allergen challenges (BAC) in moderate-to-severe asthmatics.

Identifying allergic triggers of asthma is interesting because AA patients can be treated with allergen immunotherapy (AIT). AIT is an etiologic intervention displaying a sustained clinical benefit after discontinuation and a capacity to prevent disease progression, as long as it is administered for a minimum cycle of three years [11]. In recent years, new HDM immunotherapy modalities registered as pharmaceutical products have been approved for the treatment of HDM-driven AA [12]. This review will summarize the distinct phenotypes of HDM-driven asthma, emphasize the importance of confirming the clinical relevance of immunoglobulin (Ig)E sensitizations, and discuss the many benefits associated with HDM immunotherapy in critical asthma outcomes.

## 2. Phenotyping House Dust Mite-Driven Asthma

Asthma phenotypes can be divided into those with eosinophilic bronchial inflammation (usually termed T2 asthma) as those without eosinophilic inflammation (non-T2 asthma) [4]. T2 asthma has been classically divided between AA and eosinophilic non-allergic asthma based on the presence of atopy [5]. Nevertheless, IgE sensitizations are not always clinically relevant [7]. Because >50% of asthmatics are sensitized to HDM [9], there is a need to identify bona fide allergic individuals. Moreover, new data demonstrate that some non-atopic individuals with T2 asthma can experience a positive bronchial challenge with HDM [8]. These facts demonstrate that atopy and allergy represent two different phenomena and collectively challenge the atopy-based classification of T2 asthma (Table 1).

### 2.1. Allergic Asthma

AA is characterized by the onset of typical asthma symptoms upon the exposure to one or more aeroallergens in sensitized (atopic) individuals [4]. Thus, by definition, AA patients test positive at least in one of the two classical markers of atopy: skin prick test (SPT) and allergen-specific (s)IgE in serum [5]. The relevance of HDM as triggers of AA has increased in the last decades [6], probably mirroring the global expansion of the Western lifestyle. Individuals in Western cultures spend most of their time indoors, which favors sensitization to indoor allergens [13]. In the indoor environments of coastal areas with humid and temperate climates, HDM are present year-long, yet they can experience seasonal variations [14]. Interestingly, indoor allergens are associated with more severe forms of AA as compared to outdoor pollen allergens [6]. HDMs typically induce persistent forms of AA, and a significant proportion of patients remain uncontrolled or partially controlled despite continuous inhaled therapy [6]. Besides viral infections, HDM exposure frequently triggers exacerbations in these patients, especially during the warm and humid seasons (e.g., autumn and spring) [15]. Moreover, patients with AA frequently suffer from concomitant rhinitis [3]. According to the united airways concept, allergic rhinitis (AR) and AA can be considered the organ-specific manifestations of a single chronic airway disease (atopic respiratory allergy, ARA). Of note, the onset of ARA often occurs during childhood and can persist lifelong with progressive aggravation and development of new IgE sensitizations [3].

### 2.2. Local Allergic Asthma

Recently, a new phenotype of HDM-driven asthma (LAA) has been described in individuals with local allergic rhinitis (LAR) [8]. LAR is a newly identified phenotype of chronic rhinitis characterized by the absence of atopy and positivity of the nasal allergen challenge (NAC) [16]. LAR is an independent rhinitis phenotype that does not progress to systemic atopy, although typically occurs in patients with a family history of atopy [17]. The disease often commences during childhood and progresses towards clinical worsening and asthma development [18]. In a 10-year follow-up study of 176 LAR individuals conducted by our group, the prevalence of asthma guide symptoms significantly increased from 18.8% at baseline to 30.7% at the end of the study period [19]. Similar to ARA, HDMs are the most frequent triggers of LAR [20]. These observations prompted us to evaluate the nature of bronchial symptoms in LAR patients and their relationship with allergen exposure. We recruited 28 and 18 individuals with HDM-driven LAR and AR, respectively, who also reported asthmatic symptoms [8]. Nineteen patients with non-atopic non-allergic rhinitis (NAR) suffering from concomitant asthmatic symptoms and eight healthy non-atopic control (HC) individuals were also included. All LAR and AR patients and all NAR and HC subjects had previously tested positive and negative, respectively, in a nasal challenge with HDM. Among LAR and AR patients, 28.6% and 83% displayed a positive result in the bronchial challenge with HDM, respectively, thus confirming the presence of LAA and AA. Conversely, none of the NAR and HC individuals tested positive in the BAC. Asthma was confirmed by methacholine provocation in 50%, 83%, and 58% of LAR, AR, and NAR patients, respectively, but only HDM-allergic patients experienced an increase in airway hyperresponsiveness after the BAC, regardless of their atopic status. Importantly, LAA was diagnosed in patients with LAR, which indicates that both conditions can be considered the organ-specific manifestations of a single airway disease (local respiratory allergy, LRA), and that this new phenotype also participates in the united airways concept [18]. Of note, specific reactivity to HDM is associated with eosinophilic airway inflammation in both LAR and LAA patients. On the other hand, in a recent Polish study conducted in 36 individuals with birch pollen-driven LAR, the presence of asthma and LAA was specifically investigated [21]. Of note, asthma diagnosis was confirmed in 76% of LAR patients reporting suggestive bronchial symptoms, whereas 58% of them tested positive in the bronchial challenge with birch pollen. These data illustrate that, similar to LAR, both seasonal and perennial allergens can trigger LAA.

## 3. Endotyping House Dust Mite-Driven Asthma

### 3.1. Allergic Asthma

Mouse models of HDM-driven AA showed a division of labor among antigen-presenting cells in the different phases of allergic airway inflammation. Whereas myeloid CD11b+ conventional dendritic cells were the main drivers of sensitization to HDM (by priming allergen-specific (s)Th2 cells), monocyte-derived dendritic cells behaved as the master local orchestrators during the re-challenge phase [22]. Upon allergen reencounter, massive amounts of monocytes migrate from the circulation to the bronchial mucosa, where they differentiate into inflammatory cells to release chemokines, recruit other immune cells, and locally reactivate memory sTh2 cells [22]. This labor division was later confirmed in clinical studies of AR [23] and AA [24] patients.

Primed sTh2 cells interact with naïve B cells in the secondary lymphoid tissues to induce class switch recombination to IgE (εCSR) [25]. Nevertheless, IgE-switched B cells cannot undergo efficient somatic hypermutation in the B cell follicles of germinal centers [26]. This fact determines a low frequency and insufficient affinity maturation of germinal center-derived sIgE. Conversely, IgG- and IgA-switched B cells can complete their maturation in secondary lymphoid tissues and become systemically available [25]. On the other hand, efficient IgE immune responses are preserved through the sequential εCSR of IgG_1_+ B cells in peripheral tissues [27]. Most sIgE is synthesized in AR patients through this sequential switching at the nasal mucosa after re-exposure to the allergen [28]. In AA individuals, the source of sIgE is less characterized, probably due to the greater difficulty in obtaining bronchial samples. Nevertheless, recent evidence indicates that the bronchial mucosa is a relevant site for sIgE synthesis also in allergic asthmatics [29]. Markers of εCSR and high amounts of IgE+ and high affinity receptor for IgE (FcεRI)+ cells have been identified in the bronchial mucosa of AA patients [30,31]. Moreover, a study analyzing bronchial tissue homogenates demonstrated HDM-sIgE in all AA patients included [32].

### 3.2. Local Allergic Asthma

Several studies have investigated the presence and synthesis of IgE in the airway mucosa of non-atopic individuals with rhinitis and asthma. Similar to AA, markers of εCSR and IgE+ and FcεRI+ cells have been identified in the bronchial mucosa of non-atopic eosinophilic asthmatics [29,30,31]. Similarly, sIgE+ cells were demonstrated in the nasal mucosa of non-atopic rhinitis individuals [33]. Nevertheless, there are conflicting data about the specificity and functionality of local IgE in non-atopic patients. One study detected HDM-sIgE in the sputum of 39 out of 39 non-atopic asthmatics [34]. Conversely, another work reported that HDM-sIgE was not observed in the bronchial homogenates of any of the non-atopic asthma patients analyzed [32]. In any case, none of these studies correlated the absence or presence of mucosal sIgE with the bronchial response to HDM exposure. In another work, HDM-sIgE was found in the sputum of 26 out of 27 non-atopic asthmatics, yet the patients failed to develop a positive BAC [35]. In contrast, sputum HDM-sIgE from three non-atopic asthmatics from the same series activated peripheral basophils in vitro. Nevertheless, given the heterogeneity of non-atopic rhinitis and asthma phenotypes, it seems reasonable to focus the quantification of local sIgE on those individuals with confirmed allergen-specific airway reactivity.

The pooled analysis of HDM-driven LAR individuals revealed that sIgE in the nasal secretions increases progressively during the 24 h following a positive NAC [36]. In any case, the values detected were very low, and not every patient tested positive in at least one determination. Several studies have confirmed that sIgE can only be detected in the nasal secretions of a minority (20–40%) of LAR subjects [36,37,38,39]. Although methodological factors might account for this low detection rate, it cannot be excluded that sIgE is not present in the respiratory secretions of patients with LRA [40]. Of note, individuals with LRA do not have detectable sIgE in serum, and both biological fluids are ultimately connected through the lymphoid vessels. Notably, a study using postoperative sinus sponge packs (which grow inside the nostril to perfectly adapt to the anatomy and scratch a significant amount of mucosal cells when they are removed) demonstrated that nasal sIgE is present in >90% of LAR individuals [41].

In our study defining the LAA phenotype, HDM-sIgE was not detected in the sputum of any individual experiencing a positive BAC (AA or LAA subjects) neither at baseline nor after the provocation [8]. The absence of sputum sIgE in AA patients seems to indicate that methodological aspects are at least partially related to this lack of detection [40]. The study also investigated the BAC-induced changes in tryptase, eosinophil cationic protein (ECP), T cells, natural killer (NK) cells, monocytes, and eosinophils in sputum [8]. The BAC induced a significant increase of sputum ECP, eosinophils, and monocytes in LAA and AA patients, whereas non-allergic asthma and HC subjects experienced no modification. No differences were observed for the other parameters. These findings demonstrate the allergen specificity of the inflammatory response experienced by BAC-positive individuals, regardless of their atopic status. Moreover, similar to AA [24], monocyte recruitment seems to be involved in the effector phase of LAA. Collectively, these data suggest that the immunopathology of LAA/LRA closely resembles that of AA/ARA.

## 4. Diagnosis of House Dust Mite-Driven Asthma

### 4.1. Allergic Asthma

The diagnosis of AA requires both the positivity of SPT or serum sIgE (sensitization) and the demonstration of the clinical relevance of IgE sensitizations (allergy) [4]. In atopic asthmatics with seasonal or mild persistent symptoms, the clinical history usually suffices to establish the relevance of IgE sensitizations [7]. In the case of inconclusive data, a BAC can be performed, as this test is considered the gold standard to identify the allergic triggers of asthma [42]. Nevertheless, this test is a laborious procedure, which lacks a standardized protocol for clinical use. Moreover, the BAC is not exempt from risk, thus not being recommended in patients with forced expiratory volume in the 1st second (FEV_1_) < 70% [43]. The length of the procedure is another relevant limitation. Patients who test positive in the BAC experience an early asthmatic response peaking 1–2 h after allergen inhalation [44]. Thereafter, the obstruction resolves and some, but not all, develop a late asthmatic response peaking at 7 h. The possibility of a late response determines the need for a long observation period at the hospital, sometimes including an overnight stay. Moreover, BAC protocols require the temporary withdrawal of maintenance therapy, including inhaled corticosteroids (ICSs) [43]. In patients with moderate and severe asthma, the diagnosis of AA is even more complicated. Due to the persistence and severity of symptoms, the clinical history is frequently not sufficient to establish the relevance of sensitizations [7], and many patients lose control shortly after the discontinuation of ICS, thus preventing the performance of a BAC.

One potential solution to overcome these limitations is to develop a BAC protocol that does not require the discontinuation of ICS. The occurrence of an early response establishes the positivity of the BAC, whereas the late response is the most useful parameter in research studies (e.g., to evaluate the effect of an intervention) [44]. Importantly, ICSs significantly affect the late response but have little influence over the early response [45]. This approach would be beneficial to investigate allergic triggers in moderate and severe asthma patients. On the other hand, the maintenance of ICSs might help decrease the frequency and severity of the late response [45]. Indeed, not every patient with a positive BAC will experience a late response, and the probability seems to be allergen specific (75% for HDM) [46]. In this regard, a panel of transcriptomic biomarkers able to identify patients who will develop a late response and measurable in peripheral blood has been recently identified [47]. These approaches might help personalize and shorten the BAC protocols, thus facilitating the clinical implementation of the test.

Following the united airways concept, it would be tempting to speculate that the NAC is a useful tool to phenotype the inflammatory disease affecting the airways regardless of its organ-specific manifestations (rhinitis and asthma) [48]. In our group’s study, 83% of HDM-driven AR patients (all positive for NAC by inclusion criteria) displayed a positive bronchial provocation with HDM [8]. The NAC is a safe and reproducible technique [49] counting on a validated methodology [50] and defined cutoff points for positivity [51]. Moreover, published protocols are considerably shorter than those of BAC [50,52] (Table 2).

**Figure 1:**
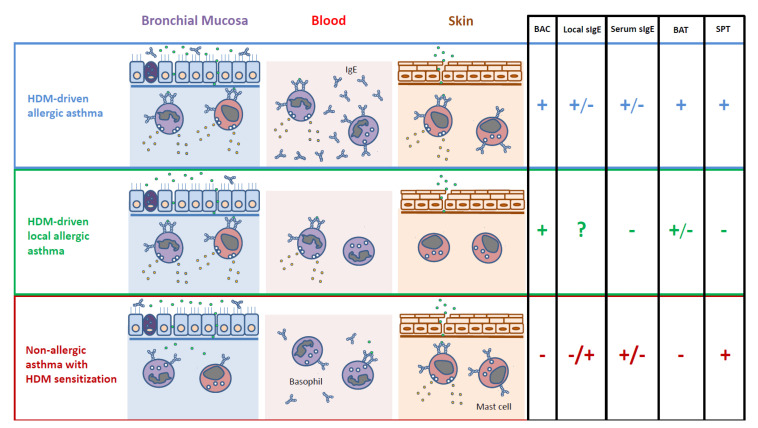
In vivo and in vitro biomarkers useful to differentiate asthma phenotypes related to sensitization and/or bronchial reactivity to house dust mites (HDMs). BAC: bronchial allergen challenge; sIgE: HDM-specific IgE; BAT: basophil activation test; SPT: skin prick test; +: positive; −: negative; ?: unknown.

The measurement of local sIgE has little diagnostic value for ARA. Besides the lack of standardized methodology, the quantification of sIgE in sputum, nasal secretions, or airway mucosa only denotes sensitization [40], and this information can be obtained through SPT/serum sIgE in a much easier manner (Figure 1). On the other hand, virtually all ARA patients display positive basophil activation test (BAT) responses with the allergens triggering their respiratory symptoms [56,57]. The BAT is considered an in vitro provocation informing not only on the presence of sIgE but also on its capacity to activate effector cells (functionality) [58]. Despite these promising aspects, no study has investigated to date the correlation between the BAT and BAC results.

### 4.2. Local Allergic Asthma

**Figure 2:**
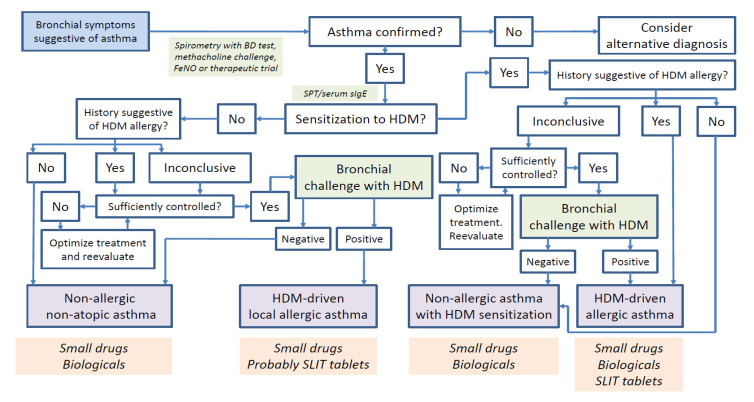
Diagnostic and therapeutic algorithm for asthma with house dust mite (HDM) sensitization and or bronchial reactivity. Blue color refers to symptoms, green color refers to diagnostic tests, purple color refers to diagnosis/phenotypes, and orange color refers to treatments. BD: bronchodilator test; FeNO: fractional exhaled nitric oxide; SPT: skin prick test; sIgE: HDM-specific IgE; SLIT: sublingual immunotherapy.

As in LAR, a BAC is required to diagnose LAA (Figure 2). The patients included in the two studies defining the LAA phenotype suffered from persistent mild-to-moderate asthma, which remained controlled through the study period [8,21]. Therefore, all individuals could be subjected to a BAC. Conversely, the relevance of LRA remains uninvestigated in severe asthmatics. Similar to ARA, it could be hypothesized that the NAC is a useful tool to phenotype LRA regardless of its organ-specific manifestations [59]. Nevertheless, in our study, only 28.6% of HDM-driven LAR patients (all positive for NAC by definition) with bronchial symptoms displayed positive BAC results [8]. Although these data question the accuracy of the NAC for LAA diagnosis, more studies and larger sample sizes are required to obtain definitive conclusions.

In LAR patients, the measurement of sIgE in the nasal secretions displays low sensitivity but high specificity (100%), implying that, when detected, nasal sIgE confirms LAR diagnosis [40]. Nevertheless, no work has investigated to date the correlation between nasal sIgE and the BAC result. On the other hand, several studies demonstrated that the sensitivity of the BAT for the diagnosis of HDM-driven LRA ranges from 50–60%, whereas the specificity is close to 100% [53,60,61]. Nevertheless, those studies were focused on rhinitis patients and yet although asthma symptoms were present in some individuals, the diagnosis of LAA was not confirmed. Unlike the quantification of sIgE in the respiratory secretions [40], BAT counts on a validated methodology and cutoff points for positivity [58], which makes this test a promising tool to facilitate LRA diagnosis.

## 5. Treatment Options for House Dust Mite-Driven Asthma

### 5.1. Small Drugs and Biologicals

Global initiative for asthma (GINA) guidelines recommend a step-based approach for treating asthma patients without consideration of the phenotype until the last treatment step [62]. ICSs are the cornerstone of maintenance treatment, and their dosage should be tailored to the patient’s severity status. From GINA step 3, different controller medications can be added, such as inhaled long-acting β2 agonists or oral leukotriene receptor antagonists, and from GINA step 4 inhaled tiotropium. Since 2019, GINA has recommended the combination of low-dose ICS-formoterol as reliever therapy for all treatment steps. GINA step 5 includes the concept of phenotypic assessment to decide on an add-on treatment, mainly biological drugs [62]. Currently, five monoclonal antibodies are recommended for the treatment of severe otherwise uncontrolled asthma: omalizumab (anti-IgE for AA with sensitization to perennial allergens), mepolizumab and reslizumab (anti-interleukin (IL)-5 for eosinophilic asthma), benralizumab (anti-IL-5Rα for eosinophilic asthma), and dupilumab (anti-IL-4Rα for T2 asthma) [63]. The indications of these five drugs are highly overlapping (e.g., in most cases, AA fulfills the criteria of T2/eosinophilic asthma), and currently, there is a lack of biomarkers able to identify differential responses when a patient fulfills indications for two or more biologicals [64]. Given the difficulties to identify bona fide allergic patients among severe asthmatics, in the clinical practice, it is usually accepted that individuals with severe uncontrolled asthma who are sensitized to HDM can receive omalizumab. Many of these patients can also be prescribed the other monoclonal antibodies, which are indicated in patients with high blood eosinophilia and fractional exhaled nitric oxide (FeNO) [65]. In any case, despite the lack of specific studies on the topic, it could be hypothesized that subjects with HDM-driven AA would respond better to omalizumab than to the other biologicals. Nevertheless, the inability to identify bona fide allergic individuals among severe asthmatics [10] prevents the confirmation of this preferential response. Conversely, LAA patients (non-atopic by definition) do not fulfill indications for omalizumab treatment [63], and no study has investigated to date the performance of this drug in subjects with LRA. Of note, the LAA phenotype has not been investigated yet among severe asthmatics.

### 5.2. Allergen Immunotherapy

AIT is the only etiological treatment for airway allergy, able to prevent disease progression (e.g., asthma onset in children with AR) and displaying a sustained beneficial effect after therapy discontinuation [66,67,68] (Table 3). AIT is also an example of precision medicine where patient selection is guided by detailed phenotyping (confirmation of sensitization and its clinical relevance), and the treatment administered selectively targets the altered immune response towards the allergen driving the symptoms [11]. AIT works via the generation of regulatory sT cells, which counterbalance the effect of sTh2 cells and promote the synthesis of sIgG_4_ [69,70]. Despite these advantages, AIT has been underused in asthma mainly due to the difficulty in identifying the disease’s allergic triggers [10] and the relative scarcity of good-quality data [68]. Of note, AIT has not been historically registered as a pharmacological product but has been considered a master formula specifically made for each patient. Nevertheless, since the early 2000s, sublingual tablets of allergen immunotherapy (SLIT tablet) registered as pharmaceutical products have been commercially available [12].

#### 5.2.1. Allergic Asthma

##### Clinical Studies

In recent years HDM-SLIT tablets (Acarizax©, ALK-Abello, Hørsholm, Denmark) at the dose of 12 SQ-HDM (standardized quality brand-specific unit denoting the biological power of the extract) were approved in Europe and the USA [71]. In a clinical trial published in 2014, 604 patients >14 years with HDM-driven AA who remained partially controlled with GINA steps 2–4 were randomized to receive HDM-SLIT tablets or placebo for 12 months [72]. Patients could continue with their standard inhaled medication, and the primary outcome was the ability of HDM-SLIT tablets to reduce the ICS dose required to maintain control. Of note, the dose of 6 SQ-HDM achieved a significant reduction of ICS (*p* = 0.004), whereas the other doses and placebo did not. At the end of the study period, >33% of patients having received 6 SQ-HDM remained controlled without daily ICS intake. The safety profile of HDM-SLIT tablets was also optimal.

In a later clinical trial published in 2016, including 834 patients >18 years with HDM-driven AA who remained partially controlled despite GINA steps 2–4, were randomized to receive HDM-SLIT tablets (either 6 or 12 SQ-HDM) or placebo for 13–18 months [73]. During the last six months of the trial, the patients were instructed to reduce the dose and discontinue ICS intake. The primary outcome was the time gap to the first exacerbation during the ICS reduction/withdrawal period. Of note, both HDM-SLIT tablet doses were associated with a significant reduction in the probability of moderate and severe exacerbations as compared to placebo, yet the effect was more prominent for the higher dose (*p* = 0.03). HDM-SLIT tablets also induced a significant increase of serum HDM-sIgG_4_ and the tolerance was excellent.

##### Positioning in Clinical Guidelines

The quality of the evidence related to the clinical effect of HDM-SLIT tablets in AA led to the inclusion of this drug as a therapeutic option in GINA guidelines since 2017. In the 2020 GINA update, the use of HDM-SLIT tablets is recommended for adult patients sensitized to HDM who have controlled or partially controlled asthma with GINA steps 3–4, concomitant AR, and FEV1 > 70% [62]. GINA recommendations established relevant novelties concerning the classical practice of AIT. Unlike other modalities, HDM-SLIT tablets can be administered to patients with sub-optimally controlled asthma or with FEV1 between 70% and 80%. Moreover, GINA resolves the issue of the clinical relevance of HDM sensitization [7] by requiring the presence of concomitant AR. Conversely, HDM-SLIT tablets are still conceived by GINA as an add-on treatment to standard inhaled therapy. GINA also favors HDM-SLIT tablets over avoidance measures as a strategy to decrease the rate of HDM-triggered exacerbations in patients with AA [2]. On the other hand, GINA does not recommend HDM-SLIT tablets in children and adolescents or in the lowest treatment steps yet. In conclusion, GINA guidelines state that the evidence provided by HDM-SLIT tablets is of considerably higher quality (grade B) than that of SLIT drops or subcutaneous immunotherapy (SCIT) (grade D).

This quality was also reflected in some national guidelines for asthma treatment. For instance, in 2020, the Spanish guidelines (*Guía Española para el Manejo del Asma*, GEMA) expanded the recommendation of AIT in adults to treatment steps 1–4 [74]. GEMA has included AIT as an accepted add-on treatment for steps 2–4 since 2009. Notably, despite acknowledging the beneficial effects of other modalities, GEMA’s 2020 update only mentions HDM-SLIT tablets. In children and adolescents, the recommendation for AIT remains for steps 2–4. Moreover, GEMA emphasizes HDM-SLIT tablets’ additional safety guarantees compared to SLIT drops and SCIT, and recommends to choose AIT modalities registered as pharmaceutical products whenever this option is available.

The European Academy of Allergy and Clinical Immunology (EAACI) guidelines for AIT in HDM-driven AA also favor SLIT tablets over SLIT drops and SCIT [75]. EAACI recommendations are mainly based on evaluating the effect of AIT modalities over four critical parameters: exacerbations, degree of control, the dose of ICS required to maintain control, and safety. HDM-SLIT tablets were the only AIT modality showing a substantial effect on the four critical parameters in adult patients with HDM-driven AA (either controlled or partially controlled). EAACI guidelines also highlight the need to differentiate between HDM-driven AA and asthma with sensitization to HDM, as only patients with the first condition are candidates for HDM-SLIT tablets [75]. According to EAACI, this differentiation might require provocation tests with HDM in some cases. Moreover, EAACI guidelines also integrate HDM-SLIT tablets in the control-based management of HDM-driven AA patients. In controlled individuals, the drug can help decrease the dose of ICS required to maintain control, whereas, in partially controlled patients, HDM-SLIT tablets might be paramount to achieving the control.

##### The Ongoing Quest for Response Biomarkers

In Japan, HDM-SLIT tablets are commercialized at a dose of 6 SQ-HDM (Miticure©, ALK-Abello, Hørsholm, Denmark) [76]. Two open parallel group studies from Japan have investigated response biomarkers using this dose. A work from 2019, including 102 patients >20 years with HDM-driven AA who remained controlled with GINA steps 2–3, investigated the effect of HDM-SLIT tablets on airway inflammation and geometry [77]. Patients were randomized to receive 6 SQ-HDM plus standard therapy or standard therapy alone for 12 months. HDM-SLIT tablets were associated with a significant decrease of FeNO and the bronchial wall thickness as measured by computerized tomography (CT) scan. Conversely, there was a significant increase in FEV1, quality of life, and airway lumen diameter. Of note, significant correlations were observed between the changes in FEV1 and FeNO. A very recent work displaying an identical design and examining a population with the same features investigated the effect of HDM-SLIT tablets on classical biomarkers of T2 asthma [78]. Of note, HDM-SLIT tablets induced a significant reduction in serum periostin, a change correlating with FEV1 reduction. The study also defined responder patients to HDM-SLIT tablets as those experiencing an increase of FEV1 greater than 120 mL and used this classification to identify cutoff points of good response for FeNO (28 ppb) and serum periostin (31 ng/mL). Interestingly, the proportion of patients testing above these limits for both biomarkers was higher in responder (44.8%) than non-responder (11.7%) individuals.

#### 5.2.2. Local Allergic Asthma

No study has investigated to date the effect of AIT on HDM-driven LAA [79]. In a clinical trial published in 2016, 36 Spanish patients with LAR due to HDM were randomized to receive either SCIT (Pangramin Plus^®^ *Dermatophagoides pteronyssinus* 1000 STU/mL, ALK-Abello, Hørsholm, Denmark) or placebo for two years [80]. SCIT administration was associated with reduced symptom and medication scores and tolerance to greater allergen concentrations in the NAC, which was significant from six months onwards. Although 28% of study individuals reported asthma guide symptoms, the diagnosis of asthma or LAA was not evaluated. Nevertheless, SCIT treatment also induced a progressive increase in serum HDM-sIgG_4_, significant from the first year. The capacity of SCIT to control nasal and conjunctival symptoms, increase the nasal tolerance to the allergen and the level of serum sIgG_4_, and to improve quality of life was demonstrated in two independent clinical trials with LAR patients due to grass pollen (Spain) and birch pollen (Poland) published in 2018 [81,82]. In the Spanish study, SCIT (Depigoid© *Phleum pratense* 1000 DPP/mL, Laboratorios Leti, Madrid, Spain) did not induce a significant difference in the bronchial symptom score measured during the grass pollen season as compared to placebo [80]. Nevertheless, SCIT had only been administered for six months at the moment of evaluation, and this study did not confirm either the diagnosis of asthma or of LAA. On the other hand, in a recent Polish clinical trial conducted in 36 LAR patients, the presence of LAA due to the birch pollen and the effect of a three-year cycle of SCIT (Alutard© *Betula verrucosa* 100,000 SQ-U/mL, ALK-Abello, Hørsholm, Denmark) on the disease were specifically analyzed [21]. BACs were performed before and after the administration period, and the bronchial tolerance to the allergen increased significantly in LAA patients treated with SCIT but not in those who received placebo.

## 6. Conclusions 

Among the different aeroallergens, those present in the feces and bodies of HDMs are related to the highest burden for patients and health systems. Their perennial presence in the coastal areas of humid and temperate regions, where most of the world population resides, makes them the most relevant agents driving airway allergy, including asthma. Moreover, HDMs are frequently related to the exacerbations experienced by subjects with moderate-to-severe AA. Nevertheless, not every asthma patient who is sensitized to HDM suffers from HDM-driven AA. The clarification of the clinical relevance of HDM sensitization is an unmet need in the clinic, especially in patients with moderate-to-severe asthma. The generation and validation of BAC protocols without ICS discontinuation might be a solution to overcome this limitation.

On the other hand, recent data indicates that HDM can trigger bronchial symptoms in non-atopic asthmatics. This novel phenotype has been termed LAA, and it can only be identified if a BAC is implemented in the diagnostic algorithms. LAA immunopathology closely resembles that of AA, including bronchial eosinophilia and monocyte recruitment. Moreover, both AA and LAA are closely related to their corresponding rhinitis phenotypes, namely AR and LAR.

The interest in identifying the allergic triggers of respiratory diseases lies on the possibility of treating them with specific immunomodulatory therapies. Unlike biologicals, AIT is an etiological and disease-modifying intervention, the clinical benefit of which persists after therapy discontinuation. New modalities of HDM immunotherapy, such as SLIT tablets, have demonstrated a robust effect on critical asthma parameters (exacerbations, the dose of ICS needed to maintain control, and safety) and are now recommended by international guidelines to treat mild-to-moderate HDM-driven AA. On the other hand, no clinical trial with AIT has been conducted in patients with HDM-driven LAA. Many other questions also remain unanswered. The effect of HDM-SLIT tablets in children and adolescents and the mildest asthma phenotypes is not established yet. Moreover, the optimal treatment duration and the long-term effect of HDM-SLIT tablets need to be investigated, as the longest trial published to date lasted only 18 months. These last two aspects remain to be established for AIT in LRA, and clinical trials specifically addressing LAA patients need to be conducted. Finally, there is a need to identify response biomarkers to AIT in both ARA and LRA patients. All these aspects are crucial steps towards a precision medicine-based management of HDM-allergic individuals, which will ultimately translate into a lower disease burden and a better quality of life for patients with HDM-driven AA and LAA (Box 1).

Box 1 **Salient points.**

House dust mites account for a significant burden of respiratory disease, including asthma exacerbations.

Allergic rhinitis and allergic asthma are organ-specific manifestations of atopic respiratory allergy, a condition defined by the positivity of skin prick test, basophil activation test, and nasal and bronchial allergen challenge. Atopic respiratory allergy is an eosinophilic inflammatory condition probably mediated by the mucosal synthesis of allergen-specific IgE.

Local allergic rhinitis and local allergic asthma are the organ-specific manifestations of local respiratory allergy, a condition defined by negativity of the skin prick test and serum allergen-specific IgE and the positivity of the basophil activation test and the nasal and bronchial allergen challenge. Local respiratory allergy is an eosinophilic inflammatory condition probably mediated by the mucosal synthesis of allergen-specific IgE.

The bronchial allergen challenge is the gold standard for the identification of the allergic triggers of asthma, both in atopic and non-atopic patients. Nevertheless, the test lacks a validated methodology for clinical use and cannot be performed in many patients with moderate-to-severe asthma.

Allergen immunotherapy is a disease-modifying treatment, the clinical benefit of which persists after therapy discontinuation. Sublingual immunotherapy with house dust mite tablets registered as a pharmaceutical product is associated with beneficial effects in several critical outcomes of allergic asthma, and is now recommended by the main international guidelines for asthma management.

## Figures and Tables

**Table 1:** Comparison of asthma phenotypes related to sensitization and/or bronchial reactivity to house dust mites.

	HDM-Driven Allergic Asthma	HDM-Driven Local Allergic Asthma	Non-Allergic Asthma with HDM Sensitization
Nasal affection	Virtually always	Always	Common, but not always
Nasal counterpart	Allergic rhinitis	Local allergic rhinitis	Non-allergic rhinitis
Atopy	Present	Absent	Present
Family history of allergy	Frequent	Frequent	Infrequent
Allergic triggers	House dust mites. Others possible.	House dust mites. Others possible.	None
Severity	Mild to severe	Only demonstrated in mild to moderate cases	Mild to severe
Age of onset	Early (childhood/adolescence)	Probably early (childhood/adolescence)	Later than allergic phenotypes
Natural evolution	Progressive worsening and onset of new systemic sensitizations	Progressive worsening and onset of new local sensitizations	Stable severity since onset in most cases
Eosinophilia	Yes	Yes	Sometimes
Bronchial sIgE	Frequent	Unknown	Possible
BAC needed for diagnosis	Sometimes	Always	Sometimes
Indication of ICS	Yes	Yes	Yes
Effect of ICS	Beneficial	Beneficial	Variable
Indication of omalizumab *	Yes	No	Theoretically not, but often prescribed **
Effect of omalizumab	Beneficial	Probably beneficial	Not beneficial in most cases
Indication of reslizumab mepolizumab, benralizumab and dupilumab *	In most cases	Potential, but the phenotype is not identified yet among severe asthmatics.	Variable
Effect of reslizumab, mepolizumab, benralizumab and dupilumab	Beneficial in most cases	Potentially beneficial, but the phenotype is not identified yet among severe asthmatics.	Variable
Indication of AIT	Yes	No	No
Effect of AIT	Beneficial	Probably beneficial	Not beneficial

HDM: house dust mite; sIgE: allergen-specific IgE; BAC: bronchial allergen challenge; ICS: inhaled corticosteroids; AIT: allergen immunotherapy; * in severe otherwise uncontrolled cases; ** See Section 4.1.

**Table 2:** Comparison of the features of the nasal and the bronchial allergen challenge performed for clinical purposes.

	Nasal Allergen Challenge	Bronchial Allergen Challenge
Standardized for clinical use	Yes	No
Need to withdraw ICS	No	Yes
Minimum FEV_1_ required	Flexible as long as the bronchial disease is sufficiently controlled	70%
Primary diagnostic use	Allergic rhinitis, local allergic rhinitis and dual allergic rhinitis [53]	Allergic asthma and local allergic asthma
Recommended monitoring system	Symptoms score (subjective) and objective measurement of the nasal patency (e.g., by acoustic rhinometry)	Bronchial obstruction by spirometry. Possible: symptom score, AHR (e.g., by methacholine challenge) and inflammation (e.g., FeNO)
Cutoff points for positivity	More defined	Less defined
Safety in asthma patients	High	Moderate
Reproducibility	High [54,55]	High
Length of the procedure including observation period	30 min to 1 h	From 7 to 24 h
Sample collection in connection to the procedure	Nasal lavage or secretions. Mucosal scraping, brushing or biopsy. FnNO.	Induced sputum, BAL. Mucosal brushing or biopsy. FeNO.
Capacity of phenotyping the united airways disease	Variable (depends on the phenotype)	Unknown

ICS: inhaled corticosteroids; AHR: airway hyperresponsiveness; BAL: bronchoalveolar lavage; FeNO: fractional exhaled nitric oxide; FnNO: fractional nasal nitric oxide; FEV_1_: forced expiratory capacity in the 1st second.

**Table 3:** Comparison of the different drug types used to treat house dust mite-driven allergic asthma.

	Small Drugs	Biologicals	Allergen Immunotherapy
Molecular weight (kDa)	0.9	150	5–50
Structure	Chemical compound	Monoclonal antibody (immunoglobulin)	Protein
Production mode	Chemical synthesis	Genetic engineering and cell culture	Purification of native extract
Site of action	Extra or intracellular	Extracellular	Extra and intracellular
Administration route	Inhaled or oral	Subcutaneous or intravenous	Subcutaneous or sublingual
Half-life	Hours	Weeks	Weeks
Dose interval	Maximum 24 h	2–4 weeks	24 h to 4–6 weeks
Precision medicine	Pharmacogenomics	Immunology and metabolomics	Molecular allergology
Specificity	Low/medium/high	High	Very high
Sustained effect after discontinuation	No	No	Yes
Disease-modifying effect	No	No	Yes
Administration period	Indefinite	Indefinite	3 years

kDa: kilodalton.

## Abbreviations

AA	allergic asthma
AHR	airway hyperresponsiveness
AIT	allergen immunotherapy
AR	allergic rhinitis
ARA	atopic respiratory allergy
BAC	bronchial allergen challenge
BAT	basophil activation test
BD test	bronchodilator test
ECP	eosinophil cationic protein
εCSR	class switch recombination to IgE
FcεRI	high affinity receptor for IgE
FeNO	fractional exhaled nitric oxide
FEV1	forced expiratory volume in the 1^st^ second
FnNO	fractional nasal nitric oxide
GINA	global initiative for asthma
HC	healthy non-atopic control
HDM	house dust mite
ICSIg	inhaled corticosteroidsimmunoglobulin
IL	interleukin
IL-4Rα	α subunit of IL-4 receptor
IL-5Rα	α subunit of IL-5 receptor
LAA	local allergic asthma
LAR	local allergic rhinitis
LRA	local respiratory allergy
NAC	nasal allergen challengenasal allergen challenge
NAR	non-allergic rhinitis
NK cell	natural killer cell
SQ-HDM	standardized quality brand-specific unit denoting the biological power of the extract
SCIT	subcutaneous immunotherapy
sIgE	allergen-specific IgE
sIgG_4_	allergen-specific IgG4
SLIT	sublingual immunotherapy
SPT	skin prick test
sT cell	allergen-specific T cell
sTh2 cell	allergen-specific Th2 cell
T2	type 2 inflammation
